# Unintended Negative Effects of the Legitimacy-Seeking Behavior of Social Enterprises on Employee Attitudes

**DOI:** 10.3389/fpsyg.2018.01991

**Published:** 2018-10-22

**Authors:** Seung Yun Lee, Donghoon Shin, Seong Hoon Park, Shomi Kim

**Affiliations:** ^1^School of Business, Konkuk University, Seoul, South Korea; ^2^The College of Business and Economics, University of Wisconsin–Whitewater, Whitewater, WI, United States; ^3^SK Corporate Contribution Team, Seoul, South Korea; ^4^Global Green Growth Institute, Seoul, South Korea

**Keywords:** social enterprises, organizational legitimacy, expected wages, employee attitudes, locus of control, psychological contract

## Abstract

In an emerging field such as social enterprise, it is important for an organization to secure legitimacy to obtain resources and sustain its business. Specifically, when a government distributing subsidies does not have adequate information to decide which organization is trustworthy, it is the legitimacy-seeking activities of a social enterprise that determines who receives a subsidy; this, in turn, decides which organization will survive. One of the most effective ways to gain legitimacy is to explicitly emphasize in the public promotion that the organization devotes to its social mission. In the case of Work Integration Social Enterprises (WISEs), an organization emphasizes its social employment of the disadvantaged individuals. However, we argue that social enterprises’ public promotion that emphasizes social employment can lower the expected wage, job satisfaction, and organizational commitment of the employees who are hired due to their disadvantaged social status. This is because such obvious promotional messages makes the employees more keenly aware of their disadvantaged status; as a result, this reinforces their self-prejudice that they are not competitive enough in the labor market. We test our hypotheses in the context of South Korean WISEs and found general support for our arguments.

“*A decent society is one in which the institutions of that society do not humiliate people*.”

- Avishai Margalit (1998)

## Introduction

As the business environment becomes more complicated and diversity increases, new forms of organizations and new fields continue to emerge and then disappear. When a field has been newly formed, its self-sustaining ecosystem (e.g., the synergistic combination of financial, commodity, and labor markets) is not fully developed, and players in the field face difficulties in finding the resources essential for their survival. Thus, they make strategic choices to attract scarce resources more effectively and consistently. Receiving the certified right to do business in the field or having themselves certified by the government is one way to obtain organizational legitimacy (Sine et al., 2007).

Recently, the social enterprise industry has attracted increasing attention. A social enterprise is an organizational form whose primary goal is to address social issues while engaging in commercial activities to generate a profit (Peattie and Morley, 2008; Battilana and Dorado, 2010; Dacin et al., 2011; Battilana et al., 2015). The social enterprise industry itself is not yet self-sustaining; social enterprises must procure necessary resources for survival outside an ecosystem that has yet to be fully developed. Because they are operating in a field where market principles cannot be fully applied, the ecosystem itself lacks diversity and necessary resources (e.g., paid customers). Therefore, it is inevitable that the majority of social enterprises depend on government subsidies.

Under this circumstance, social enterprises employ various legitimacy-seeking strategies to acquire more government subsidies. For example, social enterprises emphasize how its social employment is aligned with government job creation policy for the disadvantaged population. While this behavior can be interpreted as a natural strategy for survival, we argue that there may be unexpected, detrimental consequences from such activities on the emotional state of the beneficiaries. In this article, we elucidate how social enterprises’ promotional messages emphasizing their social employment, which are utilized to obtain legitimacy and financial support from external constituencies, may have a detrimental influence on self-esteem and attitudes (e.g., expected wage level, job satisfaction, organizational commitment, and turnover intention) of the beneficiaries.

In this study, we focused on WISEs that provide socially disadvantaged populations with employment opportunities (Peattie and Morley, 2008). Although social enterprises take various forms and utilize a wide range of modalities under the big umbrella of the social enterprise concept, one of the primary socioeconomic contributions of social enterprises lies in job creation (Battilana et al., 2015). For example, 70 percent of the Korean social enterprise is the WISE. The WISEs frequently promote their job creation for the socially disadvantaged population to demonstrate that they contribute to the government’s social employment policies. It is an essential activity for WISEs to gain legitimacy given that their resources for survival are largely dependent on government labor subsidies.

However, when beneficiaries are exposed to the explicit promotional messages of WISEs that they hire the marginalized and vulnerable groups, it may negatively affect the emotional state and attitude of the employees. An organization’s emphasis on how disadvantaged its employees are can have a negative psychological impact on them. To identify these potential impacts, we have conducted two empirical studies in which we (1) examined how a promotional message that emphasizes employing “the socially disadvantaged” affects the expected compensation of the jobseekers in social enterprises and (2) investigated the relationship between exposure to such promotional messages and consequent job attitudes of the socially disadvantaged employees.

We expect that this paper will contribute to both the theory and practice. This study contributes to the understanding of the traditional psychological contract theory by demonstrating that the conventional logic of the theory has limited applicability to the relationship between a social enterprise and its employees who are hired in the course of pursuing the SE’s social mission due to the social context surrounding them. This study also provides useful reference points to practitioners and policymakers by analyzing how the seemingly effective promotional strategies of SEs may negatively affect the targets they intend to serve, thereby suggesting how cautious they should be in designing strategies that will not weaken the foundational values social enterprises pursue.

## Legitimacy-Seeking Activity In The Emerging Field

In a newly formed industry, it is essential for organizations to acquire resources for survival. It is also important for authorities, consumers, and potential partners to determine what organizations are reliable. Therefore, obtaining legitimacy is critical for the success of an organization in an emerging industry (Aldrich and Fiol, 1994). There are two ways in which new organizations acquire legitimacy. The first is to accept the desirable norm that is assumed in the market (Baum and Oliver, 1992). The second is to take a desirable organizational form proven in the market (Aldrich and Fiol, 1994). For example, daycare centers prioritize non-profit projects rather than commercial businesses (Baum and Oliver, 1991) and enter into agreements with government agencies for financial support (Baum and Oliver, 1992). In addition, mental healthcare centers are licensed by the relevant associations (D’Aunno et al., 1991). General hospitals become members of the associations (Ruef and Scott, 1998), and volunteer groups seek to obtain a municipal registration number (Singh et al., 1986).

Legitimacy is also important for social enterprises due to the liability of their newness (Dart, 2004; Townsend and Hart, 2008; Dacin et al., 2011). These actions also help to reduce uncertainty for relevant authorities. When a new industry is formed, the authorities do not have sufficient information on which players are reliable and sustainable. Therefore, organizations which have minimal requirements are more likely to be selected to receive the support. For example, according to Zuckerman (1999), it is impossible for the authorities to compare all the organizations, and therefore, only the organizations that satisfy the legitimacy requirements of the market can become candidates for consideration.

The social enterprise industry is also a new field. In many countries, the social enterprise industry has been fostered by government policies for the recent years. For example, in South Korea, there has been a growing interest in social enterprises since the Asian financial crisis in the late 1990s (Park and Wilding, 2013). The Korean business sector became open to foreign investors and subsequently experienced massive job loss because of the financial crisis. These changes ignited public interest in social enterprises as a new model for labor opportunities. For this reason, it was government employment policy that spread the social enterprise concept across the nation, and hence, most social enterprises (e.g., WISEs) were dependent on state funding for their very existence from their inception (McCabe and Hahn, 2006). More specifically, The Ministry of Labor in South Korea has attempted to grant subsidies to social enterprises in an effort to encourage the establishment and continuance of social employment. Although social enterprises need to fulfill demanding eligibility criteria in order to receive subsidies from the government, they can obtain a secure income stream for their labor costs once they meet the criteria (Park and Wilding, 2013). In addition, the government has the right to approve the establishment and registration of social enterprises. Due to this dependence on the government, social enterprises in South Korea actively engage in legitimacy-seeking activities.

Legitimacy is also important for social enterprises due to the liability of their newness (Dart, 2004; Townsend and Hart, 2008; Dacin et al., 2011). For the government, which has the authority to allocate subsidies, it is important to identify players who are more likely to implement government policies. Under the circumstance where social enterprises have only a precarious status, constrained by the nature of a new domain, they are prompted to seek legitimacy for their existence to be selected by the government, which is the only means for assured survival. For this reason, the more social enterprises strive to earn the legitimacy, the more likely they are to receive support funds. Therefore, social enterprises try to publicize their social missions and corresponding activities, such as providing jobs for the socially disadvantaged, through means of advertisements, public relations and promotional messages (Allan, 2005). For example, some WISEs actively promote on their websites how well-aligned they are with the government’s social employment policies.

## Inadvertent Negative Impact Of Promotional Messages On Beneficiaries

Due to their quest for legitimacy, social enterprises are inclined to promote their contributions to society, particularly by emphasizing how they are creating social employment to show they fit well with government policies. In this case, successfully obtaining legitimacy would determine how much financial resources they could secure. This may encourage social enterprises to use more aggressive communication methods, such as using in their websites and publications with graphical statistics about social employment, pictures of marginalized and vulnerable employees, and/or quotes from interviews with socially disadvantaged employees. We introduce two publicity examples in the Appendix. Both companies introduced here are WISEs.

Generally, the socially beneficial activities of an organization positively influence the emotions of employees (Yoon et al., 2006; Turker, 2008). However, in the case of social enterprises, we argue that these explicit promotions may have an inadvertent negative impact on the emotional state of marginalized and vulnerable groups. This is because socially disadvantaged employees may regard themselves as charity cases for the organizational policy, rather than as worthy employees, even when they positively consider the job opportunity itself. Elliott (1996) showed that being the beneficiary of welfare policies had consistently decreased the self-esteem of female recipients over time. Based on this finding, we hypothesized the negative psychological impact of social enterprises’ promotion that emphasizes that they hire the disadvantaged individuals on two fronts: expected wages and employee attitude.

### Expected Wages

Wage expectations reflect how people make judgments on their social status (Solow, 1990). For example, expected wages demonstrate how jobseekers perceive their status in the labor market. Although various factors affect the expected wage (Brunello et al., 2004), psychological variables and emotional states have a particularly large impact on such expectations. For instance, Maani and Studenmund (1986) argued that wage expectations are largely dependent on the perception of jobseekers in the actual job market situation. This relationship can be explained by the theory of locus of control. The locus of control is a generalized attitude and belief regarding the nature of the causal relationship between one’s own behavior and its consequences (Rotter, 1966). Those believing their outcomes are due to their own efforts have an internal locus of control, while those believing outcomes are due to external factors are defined as having an external locus of control (Cobb-Clark, 2015).

Locus of control affects the expected wage in two ways: direct adjustment of the wage and indirect determination of it through self-esteem (Goldsmith et al., 1997). According to Coleman and DeLeire (2003), American adolescents with an internal locus of control anticipate higher wage returns. McGee (2015) also found that the young unemployed individuals with an internal locus of control set a higher expected wage than those with an external locus of control. From these studies, we can infer that jobseekers with an external locus of control have a lower expected wage.

The locus of control also influences self-esteem, which, in turn, affects expected wages (Goldsmith et al., 1997). Individuals who think they are not masters of their own fates are called externalizers by psychologists. Externalizers who doubt their personal efficacy are less likely to possess strong self-esteem relative to internalizers. In addition, people with low self-esteem are likely to have low expected wages. The locus of control is also influenced by previous labor market experiences (Goldsmith et al., 1996). When socially disadvantaged jobseekers are exposed to the message of a social enterprise that the company hires “the underprivileged,” they will become more aware of their vulnerable position in the labor market, and thus, are more likely to have an external locus of control. Then, their reinforced or newly adopted external locus of control directly lowers their expected wages or indirectly does so by lowering their already low self-esteem.

In addition, people tend to align their wage expectations to other individuals with whom they feel a similarity (Paola et al., 2005). If a certain jobseeker believes that (s)he has a social status, educational or professional experience similar to others, (s)he is likely to expect a similar wage level. We expect that when marginalized and vulnerable jobseekers are exposed to the promotional messages of their company’s social employment, they will associate themselves with the vulnerable and helpless beneficiaries portrayed in the promotional message, and consequently lower their expected wages, believing they are as vulnerable as those living on welfare. Based on this, we suggest the following research model and hypothesis:

**H1:** Exposure to a social enterprise’s public promotion regarding its hiring of the socially disadvantaged will lower their expected wages in the focal social enterprise.

### Employee Attitudes

When marginalized and vulnerable employees discover their employment was used to promote the company, they may feel that they have been exploited by the company. According to the psychological contract theory, this situation is a breach of an implicit contract.

A psychological contract is the belief of employees regarding a reciprocal obligation between themselves and their organizations (Rousseau, 1989). This obligation is established not only through a formal contract, but also through means that are more implicit (Morrison and Robinson, 1997). When an organization is perceived to have failed to fulfill its obligation, it is deemed to have breached the psychological contract (Morrison and Robinson, 1997). This psychological contract breach can result not only from the failure to fulfill the actual obligation, but also from the employees’ psychological perception of implicit contract breach (Fayyazi and Aslani, 2015).

For example, cognitive incongruence between an employee and his/her organization can also cause a breach of the psychological contract. Due to the hybridity of a social enterprise which simultaneously pursues business objectives and social missions (Dart, 2004; Kraatz and Block, 2008; Townsend and Hart, 2008; Dacin et al., 2011; Jay, 2013; Pache and Santos, 2013; Battilana and Lee, 2014; Battilana et al., 2015; Mongelli et al., 2017), there is a possibility that the perception of social employment can vary widely between vulnerable employees and entrepreneurs. While employees from the vulnerable class may view social employment as a benefit that should be given to them, some entrepreneurs may view it only as a business for the survival of a WISE. The perception of their company using employees only for business can also be a cause for a psychological contract breach.

According to a meta-analysis by Zhao et al. (2007), a psychological contract breach is negatively related to the attitude of employees in areas such as job satisfaction, organizational commitment, and turnover intention. We thought that in the situation of a contract breach, the consequent attitude of marginalized and vulnerable groups would differ from those that are not in such contract breach situation. Although a psychological contract breach may have the same negative impact on job satisfaction and organizational commitment of the socially disadvantaged employees as it would for regular employees, the socially disadvantaged are less likely to leave their workplaces than are other employees. This means that a psychological contract breach would not prompt them to consider leaving their organizations, unlike other regular employees, even when socially disadvantaged employees feel offended by being treated as charity case since they lack alternative job opportunities.

According to Morrison and Robinson’s comprehensive psychological contract violation model (Morrison and Robinson, 1997), employee attitudes are affected by three factors: perceived costs, self-serving biases, and a threshold. Regarding perceived cost, employees who perceive the cost of revealing the breach would be higher respond less emotionally to the breach. In other words, those who feel that they may be threatened or harmed if they expose a contract breach become less emotional about that breach. Morrison and Robinson (1997) found that employees with low self-esteem particularly tend to behave in this manner. Regarding self-serving biases, employees who think they receive more from the organization than they contribute to it and employees with low self-esteem are less emotional about a breach situation (Morrison and Robinson, 1997).

Lastly, regarding a threshold, the height of employees’ threshold affects their perception of a breach of contract and affective violation (Morrison and Robinson, 1997). Employees with higher thresholds are less likely to perceive a breach of contract than those with lower thresholds. They are therefore less sensitive to a violation. The height of a threshold is decided by the power asymmetry between employees and their organizations (Morrison and Robinson, 1997). Less powerful employees who do not feel entitled to more benefits are apt to have a higher threshold. Marginalized and vulnerable groups in the labor market who perceive themselves as less powerful than organizations are likely to have a higher threshold. Therefore, socially disadvantaged employees with higher thresholds become less disgruntled toward their status even when they perceive that their organizations committed a contract breach.

Thus, we infer that even though job satisfaction and organizational commitment can be eroded by a breach of contract, the turnover intention of social enterprise employees does not increase because marginalized and vulnerable employees believe they are less likely to be hired by another company. Although previous research indicated turnover intention is less constrained by exogenous factors such as availability of an alternative job (Zhao et al., 2007), we expect that vulnerable employees are much less likely to develop turnover intention compared to regular employees. Turnover intention is affected by emotions such as job satisfaction (Tett and Meyer, 1993) and the linkage between job satisfaction and turnover intention is mediated by thoughts of quitting and intent to search for alternative employment (Mobley, 1977). Thus, we expect that promotional messages emphasizing the social mission of saving “the underprivileged” would discourage the vulnerable employee from looking for better jobs, make them more keenly aware of their disadvantaged status, and, as a result, reinforce their self-prejudice that they are not competitive enough to search for an alternative job. In other words, even when they feel defrauded, vulnerable employees are not likely to act on their desire to get a better job. They are more likely to remain in the social enterprise which “generously” gave them a job, regardless of their emotional attitude toward the company. In this regard, we suggest the following research model and hypothesis:

**H2:** Public promotion of social enterprise which emphasizes hiring of the socially disadvantaged will:
lower job satisfaction, (H2a)
  • lower organizational commitment of a vulnerable employee in the focal social enterprise, (H2b)
  • but this will not translate into an increase of turnover intention (H2c).

**FIGURE 1:**
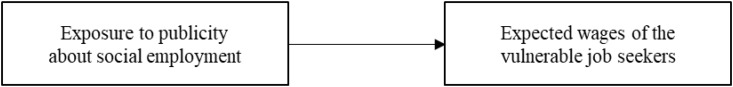
Research model 1.

**FIGURE 2:**
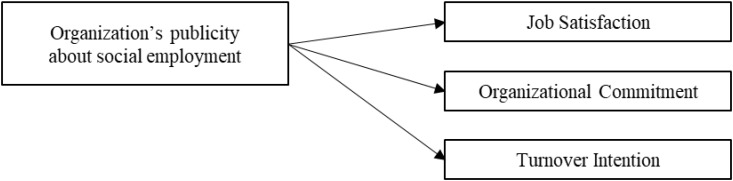
Research model 2.

Our proposed model is shown in Figures 1, 2.

## Study 1

All studies (study 1 and study 2) in this paper were carried out in accordance with the recommendations of the Research Ethics Declaration of Helsinki.

### Methods

#### Participants and Procedures

Study 1 was designed as a quasi-experiment. A total of 158 socially disadvantaged jobseekers living in South Korea participated in this experiment. They were recruited through one local government office in Seoul. The characteristics of the participants are presented in Table 1.

**Table 1:** Characteristics of participants (study 1).

Gender	Female	61%
	Male	39%
Age	Below 50	25%
	In the 50s	35%
	Over 60	40%
Education level	Elementary school graduates	26%
	Middle school graduates	51%
	High school graduates	23%


***N* = 158.**

Participants were told that the purpose of the study was to understand jobseekers’ response to a new social enterprise. Participants were randomly grouped into two categories, based on the types of promotional message. Participants were then told that they would be shown a portion of the website featuring the introductory information for the company. The existence of the social enterprise’s public promotion regarding its hiring of the socially disadvantaged was manipulated. Participants in the public promotion absent condition (Group 1) were shown the following information: “This social enterprise is committed to environmental protection for the next generation to come.” Conversely, participants in the public promotion present condition (Group 2) were shown the following information: “This social enterprise is committed to finding better jobs for the socially disadvantaged and is simultaneously committed to environmental protection.” Following these manipulations, participants were asked to imagine that they were given a job offer at the social enterprise, and that they should try to decide an amount for their expected monthly wages.

#### Measures

We measured expected wages, given that pay level is a critical indicator of self-esteem, because there is a significant linear relationship between the two variables (Thierry, 2001; Gardner et al., 2004). There are several factors that influence one’s expected wages: length of unemployment; individual aspects, such as educational background; skill level, etc. (Prasad, 2003). Thus, we controlled for participants’ length of unemployment, number of job search failures, educational background, skill level, age, and gender.

### Results

Table 2 presents descriptive statistics and correlations among variables for Study 1. Generally, the longer the unemployment period for a participant, the lower the expected wage. However, responses from participants in this study did not demonstrate a negative relationship between the unemployment period and the expected wage. Perhaps this was because these participants were beneficiaries of the same local government’s job assistance program, so the gap between their unemployment periods was not much greater than 1 year. However, the higher the number of failed job searches and the higher the age, the lower the expected wage became. In addition, the higher the levels of education and skill were, the higher the expected wage became. These correlations were consistent with the results of previous studies on expected wages (Prasad, 2003).

**Table 2:** Means, standard deviations, and correlations of variables (Study 1).

Variables	*M*	*SD*	1	2	3	4	5	6	7
(1) Expected wage	113.32	31.772							
(2) Length of unemployment	9.11	7.787	0.105						
(3) No. of failure in job search	2.26	1.636	-0.336***	0.011					
(4) Gender	0.61	0.490	-0.742***	0.026	0.295***				
(5) Age	55.41	8.675	-0.494***	0.049	0.144*	0.309***			
(6) Education	1.97	0.700	0.559***	0.204**	-0.204**	-0.464***	-0.245***		
(7) Skill level	0.06	0.233	0.509***	-0.060	-0.173**	-0.306***	-0.270***	0.364***	
(8) Public promotion	0.54	0.500	-0.777***	-0.012	0.241***	0.841***	0.355***	-0.388***	-0.265***


***N* = 158. Expected wage was measured in Korean 10,000 won. Length of unemployment was measured in months. Gender was coded as 0 = male and 1 = female. Education was coded as 1 = Elementary school graduation, 2 = Middle school graduation, 3 = High school graduation, 4 = College graduation, 5 = More than a master’s degree. Skill level was coded as 0 = Non-certification and 1 = Hold certification. Public promotion was coded as 0 = Non-existence of the SE’s public promotion regarding its hiring of the socially disadvantaged and 1 = Existence of the SE’s public promotion regarding its hiring of the socially disadvantaged. ^∗^*p* < 0.05. ^∗∗^*p* < 0.01. ^∗∗∗^*p* < 0.001.**

Hypothesis 1 predicts that exposure to public promotion of social employment will lower the expected wages of marginalized and vulnerable jobseekers. The results of the hierarchical regression in Table 3 support HI and show that there was a significant negative relationship between exposure to public promotion of social employment and the expected wage (Adjusted β = -0.447, *p* < 0.001).

**Table 3:** Summary of hierarchical regression analysis for variables predicting expected wages (Study 1).

Variables	Expected Wage (Control Model)	Expected Wage (H1)
	Adjusted β	Adjusted β
**Control variables**		
Length of unemployment	0.116**	0.097*
No. of failure in job search	-0.084 +	-0.089*
Gender	-0.514***	-0.145 +
Age	-0.232***	-0.182***
Education	0.139**	0.152***
Skill level	0.231***	0.232***
**Independent variable**		
Public promotion		-0.447***
Adjusted *R*^2^	0.721***	0.778***
Δ *R*^2^		0.056***


***N* = 158. ^+^*p* < 0.1. ^∗^*p* < 0.05. ^∗∗^*p* < 0.01. ^∗∗∗^*p* < 0.001.**

In the additional one-way analysis of covariance (ANCOVA) results [*F*(1,150) = 39.58, *p* < 0.001] showed that participants in the absent condition of the public promotion (Group 1) expected higher monthly wages than those in the present condition of the public promotion (Group 2) [*M* = 1,300 $ (Group 1) vs. *M* = 820 $ (Group 2), *t*(156) = 15.40, *p* < 0.001].

## Study 2

### Methods

#### Participants and Procedures

In study 2, a total of 177 socially disadvantaged employees working in social enterprises participated. The participants were employed by 15 different social enterprises in South Korea. These participants were randomly grouped into two categories, based on the types of promotional message publicized on the website of each social enterprise. The first group, (made up of 7 companies, 104 participants, coded as (1) was composed of employees from the social enterprises that publicly promoted their contribution of social employment; for instance one website stated, “Our mission is to create decent jobs for *the marginalized groups*”; “We are committed to a better life for the marginalized, disabled, and elderly by giving them job opportunities”; and “We have created 100 social jobs for marginalized groups since 2007.” The remaining eight social enterprises were classified into the second group with 73 participants (coded as 0). The social enterprises in this latter group did not use such promotional messages on their websites. The characteristics of SEs and employees are presented in Table 4.

**Table 4:** Characteristics of social enterprises and their employees (Study 2).

Characteristics of SEs	Type of organization	NPO	44%
		Corporation	56%
	Type of Social Mission	Social Service Delivery	18.1%
		Social Employment	46.3%
		Mixed	35.6%
	Industry	Care	37.3%
		Education	5.1%
		Health	4.5%
		Service	29.4%
		Food	11.3%
		Agriculture	12.4%
Characteristics of Employees	Gender	Female	75%
		Male	25%
	Age	Below 40	23.7%
		In the 40s	28.8%
		In the 50s	25.5%
		Over 60	22%
	Type of Contract	Non-permanent	62.7%
		Part-time	9.6%
		Permanent	27.7%
	Type of the socially disadvantaged	The age	11.3%
		Unmarried mom	6.8%
		The disabled	4%
		The poor	27.1%
		Others	50.8%
	Job	Line	84.2%
		Staff	15.8%


***N* = 177.**

#### Measures

Job satisfaction of each employee was measured by four items of a short version of a job satisfaction scale (Brayfield and Rothe, 1951; Rafferty and Griffin, 2009). The Cronbach’s α was 0.852. Organizational commitment of each employee was measured by 5 items of the affective organizational commitment scale from Meyer et al. (1993)’s study The Cronbach’s α was 0.744. Turnover intention was measured by 3 items from a withdrawal intention scale used in the Hanisch and Hulin (1991)’s study. The Cronbach’s α was 0.781. All items were measured with a 5-point Likert scale (1 = Strongly disagree; 5 = Strongly agree). Additionally, gender, age, contract type of employees, and industry type of the companies were controlled for.

### Results

Table 5 presents descriptive statistics and correlations between variables for Study 2. Job satisfaction had a positive correlation with organizational commitment and a negative correlation with turnover intention. Organizational commitment also had a negative correlation with turnover intention. Public promotion had negative correlations with job satisfaction and organizational commitment and a positive correlation with turnover intention.

**Table 5:** Means, standard deviations, and correlations of variables (Study 2).

Variables	*M*	*SD*	1	2	3	4	5	6	7	8	9	10	11	12
(1) Job satisfaction	3.55	0.80												
(2) Organizational commitment	3.47	0.62	0.634***											
(3) Turnover intention	2.69	0.95	-0.510***	-0.322***										
(4) Age	2.45	1.08	0.206**	0.070	-0.261***									
(5) Gender	0.75	0.43	-0.002	0.106	0.014	-0.204***								
(6) Type of employment	0.48	0.58	0.065	0.142*	0.007	-0.302***	-0.023							
(7) Type of work	0.84	0.36	0.014	-0.031	-0.127	0.471***	0.001	-0.317***						
(8) Industry dummy_Education	0.05	0.22	-0.020	-0.009	0.110	-0.194***	-0.045	0.149*	-0.393***					
(9) Industry dummy_Healthcare	0.04	0.20	0.054	0.115	-0.112	-0.193***	-0.001	0.306***	-0.204**	-0.050				
(10) Industry dummy_Service	0.29	0.45	0.068	0.170*	0.080	0.060	-0.002	0.034	-0.094	-0.149	-0.140*			
(11) Industry dummy_Food	0.11	0.31	0.141***	0.120	-0.211***	0.047	-0.001	0.104	0.106	-0.083	-0.078	-0.230***		
(12) Industry dummy_Agriculture	0.12	0.33	-0.102	-0.152*	0.024	0.110	-0.417***	0.030	-0.024	-0.087	-0.082	-0.243***	-0.134*	
(13) Pubic promotion	0.58	0.49	-0.227***	-0.299***	0.143*	0.015	0.023	-0.055	0.203**	0.194**	-0.260***	-0.493***	-0.426***	0.176**


***N* = 177. Gender was coded as 0 = male and 1 = female. Type of employment was coded as 0 = Part time, 1 = Full time. Type of work was coded as 0 = Staff, 1 = Line. Public promotion was coded as 0 = Non-existence of the SE’s public promotion regarding its hiring of the socially disadvantaged and 1 = Existence of the SE’s public promotion regarding its hiring of the socially disadvantaged. ^∗^*p* < 0.05. ^∗∗^*p* < 0.01. ^∗∗∗^*p* < 0.001.**

Hypothesis 2 predicts that a social enterprise’s public promotion which emphasizes its hiring of the socially disadvantaged (H2a) will lower job satisfaction and (H2b) organizational commitment of a vulnerable employee in the focal social enterprise, (H2c) but that this is not translated into an increase of turnover intention. Table 6 shows the results of the hierarchical regression that was used to test this hypothesis. Supporting H2a, there is a significant negative relationship between an organization’s public promotion about social employment and job satisfaction (Adjusted β = -0.405, *p* < 0.01). Supporting H2b, there was a significant negative relationship between an organization’s public promotion about social employment and the organizational commitment of a vulnerable employee (Adjusted β = -0.393, *p* < 0.05). Supporting H2c, there was no significant relationship between an organization’s public promotion of social employment and turnover intention (Adjusted β = 0.126, n.s.). Therefore, all three sub-hypotheses of hypothesis 2 were supported. This demonstrates that the promotion of social employment by the social enterprise lowers emotional job satisfaction and the organizational commitment of vulnerable employees. However, these effects did not increase the turnover intentions.

**Table 6:** Summary of hierarchical regression analysis for variables predicting employee attitudes (Study 2).

Variables	Job satisfaction (H2a)	Organizational commitment (H2b)	Turnover intention (H2c)
			
	Adjusted β	Adjusted β	Adjusted β
**Control variables**			
Age	0.295***	0.159	-0.301***
Gender	0.009	0.121	-0.036
Type of employment	0.188*	0.206*	-0.047
Type of work	-0.009	0.063	-0.009
Industry dummy_Education	0.019	0.069	0.038
Industry dummy_Healthcare	-0.109	-0.028	-0.110
Industry dummy_Service	-0.239	-0.073	0.134
Industry dummy_Food	-0.146	-0.107	-0.108
Industry dummy_Agriculture	-0.150	-0.083	0.033
**Independent variable**			
Public promotion	-0.405**	-0.393*	0.126
**Adjusted *R*^2^**	0.85**	0.101***	0.96**


***N* = 177. ^+^*p* < 0.1. ^∗^*p* < 0.05. ^∗∗^*p* < 0.01. ^∗∗∗^*p* < 0.001.**

## General Discussion

This paper illuminates how an institutionalized activity of social enterprises can have an inadvertent negative impact on their beneficiaries, thereby diluting their *raison d’être*. In an emerging field including that of social enterprise, a legitimacy-seeking strategy is essential since the organizations need to procure resources outside the field for survival. Therefore, organizations in such domains need to promote how well their activities fit with the intent of a key actor that holds the resources (e.g., the government). When they experience increases in external support with this publicity strategy, they become increasingly active in accentuating their message.

However, if such publicity is used as a survival strategy and not properly designed, it can have unexpected negative consequences. As predicted in this study, when jobseekers were exposed to promotional messages emphasizing social employment, their self-esteem was damaged, and this led to lower wage expectation of the socially disadvantaged employees due to their perception of external locus of control. Additionally, the more their social enterprise promotes social employment, the lower the job satisfaction and organizational commitment of vulnerable employees became due to the psychological contract breach. However, the promotion of social employment did not negatively affect the turnover intention of vulnerable employees, because they became more sensitive to their vulnerable status in the labor market.

This paper contributes to the existing literature on traditional psychological contract theory. We inferred that a psychological contract breach could occur even when vulnerable employees had a different perception of social employment than do entrepreneurs. This gap is created by the hybridity of a social enterprise. In addition, this paper shows that the vulnerable employees’ change of attitudes in the situation of psychological contract breach may be different from that of ordinary employees. As a result, employees from vulnerable groups may not have higher turnover intention even in emotionally hurtful situations.

Our paper also provides practical implications to social entrepreneurs and policymakers. It is meaningful to illuminate the unexpected secondary effect of the legitimacy-seeking behavior through the study. Social entrepreneurs need to carefully design organizational practices in order to avoid adversely influencing their beneficiaries’ emotions. Although securing a job opportunity is important for the beneficiaries of social enterprises, such beneficiaries want to be respected as human beings with dignity and treated fairly, just like other employees. Like other employees, the beneficiaries of social enterprises will be more committed to organizational activities and will contribute more when they feel they are valued members of the focal social enterprises. It is important for social entrepreneurs to empower the beneficiaries of social enterprises to be more proactive and participative (Rimac et al., 2012; Mongelli et al., 2016). Thus, if social entrepreneurs remain cognizant of this while carefully designing promotional materials, they can pursue their social mandate and secure resources synergistically without dismantling each other.

Relatedly, our results elucidate the ethical issues of social entrepreneurs. As suggested by Longnecker et al. (1988), social entrepreneurs who are equipped with strong egoism and a sense of calling may believe that the prosocial values should be accomplished by any means (and even by neglecting the appropriate procedures). However, Maak and Stoetter (2012) showed that ethical leadership is a critical element for the long-term success of social enterprises, particularly when trust levels are low. As presented by our results, neglecting the emotional states of beneficiary-employees of social enterprises can create negative attitudes among employees. This is not conducive to the long-term success and survival of social enterprises, since member commitment and loyalty are critical assets for any organization. Thus, social entrepreneurs should attend to the details of their practice and their organizing practices in pursuing valuable social missions.

### Future Research Opportunities

There are several interesting avenues for future research. We believe a more in-depth understanding of moderating variables will strengthen our knowledge on attitudes of vulnerable employees. Due to the difficulty in obtaining survey data from the beneficiary-employees of social enterprises, we obtained a somewhat limited variety of micro-level variables. This indicates that our study needs an analysis of the various control or moderating variables that affect the emotional attitudes of employees.

Second, the use of qualitative data would benefit our knowledge. In this study, we have not been able to describe the exact emotions the vulnerable jobseekers and employees felt. Such data can be obtained through in-depth interviews with participants. The demographic data also have important implications. For example, in the post-analysis of study 1, we found that the education level of jobseekers moderated the effect of publicity exposure on expected wages (Adjusted β of moderating variable = 0.342, *p* < 0.01, Adjusted *R*^2^ = 0.786). Specifically, in the situation of an exposure to publicity about social employment, highly educated people had relatively lower expected wages. Perhaps this is because highly educated people are more sensitive to and pessimistic about these promotions than are less educated people. These results are very intriguing, but we could not analyze them more deeply because discovering their cause was not the original intent of this study. In addition, we do not know which ethnographic factors most influenced the emotions of the vulnerable employee. It would be practically and theoretically meaningful to analyze what cultural factors affect the beliefs and behaviors of the socially disadvantaged employee.

Third, we need to delve further into how social enterprises’ legitimacy-seeking activities affect the behavior of employees and the performance of organizations. This study focused on the emotional changes of the socially disadvantaged employees. Researchers have found that a perceived contract breach and the resulting violation of employees reduce their contributions to their organizations (Robinson et al., 1994; Robinson and Rousseau, 1994), have a negative impact on extra-role or organizational citizenship behaviors (Robinson and Morrison, 1995), and may damage organizations’ external reputations (McLean Parks and Schmedemann, 1994). Improperly designed legitimacy-seeking behaviors of social enterprises can have a negative impact on the behavior of their marginalized and vulnerable employees. This can also have a negative impact on the performance of the company. Since vulnerable employees may be the core asset of an enterprise, this can have a negative impact on corporate competitiveness, or undesirable behaviors of the vulnerable employee may have a negative impact on the behavior of regular employees within the company.

Fourth, we also need to study the factors that reduce these negative effects. According to the comprehensive model of psychological contract violation from Morrison and Robinson (1997), trust in the organization, procedural fairness, and interactional justice lower the likelihood of violation. In particular, it would be meaningful to discover the positive factors that can be applied specifically to social enterprises. For example, social entrepreneurs’ sense of calling, sharing of social mission, and democratic decision-making processes may reduce the negative effect of a psychological contract breach.

Fifth, research on other types of social enterprises beyond WISEs and other types of employees is needed. There are various types of social enterprises addressing various social problems such as environment, health, etc., and some of them do not limit their jobs to socially vulnerable people. These social enterprises also must rely on external resources to survive because of the inherent constraints of solving social problems in areas where markets fail. Thus, just as WISEs use their social employment performance to promote themselves, they need their own legitimacy-seeking strategies. Regarding this, it would also be meaningful to discover new unintended negative effects of their legitimacy-seeking activities. In addition, research on other types of employees not categorized as the vulnerable is needed. There are various types of employees in social enterprises besides marginalized and vulnerable people. For example, in the social enterprise, there are also highly qualified and well-educated millennials. They prefer socially meaningful work, want to have fun at work, and do not hesitate to move to another job that better fits these requirements. Unsurprisingly, their reaction and subsequent actions would be different when they face the improperly designed legitimacy-seeking activities. Therefore, this factor should also be studied and included when designing the publicity model for survival.

Finally, the generalizability of our research context is also worth mentioning. Although some contextual factors of South Korea may have an impact on the interpretation of our outcomes (Park and Wilding, 2013), we hope that our outcomes can be applied to other fields where a legitimacy-seeking strategy is needed. For example, startups in new industries or new departments of government entities often lack internal resources and thus rely on external resources. This makes them pursue legitimacy-seeking strategies for survival and would subject them to a non-systematic approach. This study, which has shown the unexpected negative effects of a survival strategy when it is not structured carefully with various stakeholders in mind, can serve as a good reference point for those new entities.

## Ethics Statement

All procedures performed in studies involving human participants were in accordance with the ethical standards of the Institutional Research Committee (Yonsei University) and with the 1964 Helsinki declaration and its later amendments or comparable ethical standards. Informed consent was obtained from all individual participants included in the study. Once the surveys had been completed, researchers provided participants with accurate and appropriate information about the nature of the study.

## Author Contributions

All authors substantially contributed to the conception and the design of the work as well as in the analyses and interpretation of the data. SL prepared the draft. DS reviewed it critically and gave important intellectual input. SP and SK worked for the final approval of the version that should be published.

## Conflict of Interest Statement

The authors declare that the research was conducted in the absence of any commercial or financial relationships that could be construed as a potential conflict of interest.

## Appendix

The picture below is from a webpage of a social enterprise that hires elderly people to make boxed lunches and deliver them to children in vulnerable classes free of charge. On their web pages, there is no publicity of elderly employees and children in the vulnerable class. They only promote the quality and nutritional merits of the boxed lunches.

The picture below captures a still from a publicity video on a web page of a social enterprise that hires people with intellectual disabilities to make hats. This video depicts an event in which one of the former presidents of South Korea visits the company and was directed to the work place intentionally. The Korean text in the upper right corner is promotional text and states, “The president is visiting!”

Below is a picture of a couple with disabilities abruptly interviewed by the president. They stated that the company has given them jobs, so they could raise a baby.
